# School Suspension as a Predictor of Young Adult Homelessness: The International Youth Development Study

**DOI:** 10.1007/s10935-025-00829-y

**Published:** 2025-02-16

**Authors:** Jessica A. Heerde, Jennifer A. Bailey, Gabriel J. Merrin, Monika Raniti, George C. Patton, John W. Toumbourou, Susan M. Sawyer

**Affiliations:** 1 Department of Paediatrics, The University of Melbourne, Royal Children’s Hospital Campus, 50 Flemington Road, Parkville, VIC 3052 Australia; 2 Department of Social Work, The University of Melbourne, Parkville, Australia; 3 Centre for Adolescent Health, Royal Children’s Hospital, Parkville, Australia; 4 Murdoch Children’s Research Institute, Parkville, Australia; 5 School of Population Health, Curtin University, Perth, Australia; 6 Social Development Research Group, School of Social Work, University of Washington, Seattle, USA; 7 Department of Human Development and Family Science, Syracuse University, Syracuse, USA; 8 Centre for Social and Early Emotional Development, School of Psychology, Deakin University, Geelong, Australia

**Keywords:** Homelessness, Adolescents, Young adults, Risk and protective factors, Longitudinal, Prevention

## Abstract

School suspension in adolescence has been shown to predict homelessness in young adulthood, suggesting that it may be a point of intervention to reduce young adult homelessness. Under zero tolerance policies, school suspension is more common in the United States relative to Australia. Multilevel modeling of cross-national longitudinal data from the International Youth Development Study tested prospective associations between adolescent problem behaviors, student-perceived likelihood of suspension/expulsion, school-level behavior management policy, and young adult homelessness. Population-based samples of participants from Washington State (United States) and Victoria (Australia) were surveyed at ages 13, 14, 15 (2002–2004), and 25 years (2014–15; n = 1945; 51% female). Over half of the young adults who reported homelessness within the previous year at age 25 had experienced school suspension. Individual-level school suspension in middle school predicted young adult homelessness. Higher levels of adolescent rebelliousness, non-violent and violent antisocial behavior, and substance use predicted a higher likelihood of school suspension at the person-level and were indirectly related to increased risk for homelessness at age 25. School behavior management policy was not related to a history of school suspension at either the person- or school-level once individual factors were controlled. Findings demonstrate the importance of school suspension as a risk factor for future homelessness and suggest that prevention programming that aims to mitigate substance use, antisocial behaviors, and school suspension may help to reduce young adult homelessness.

**Supplementary Information:**

The online version contains supplementary material available at 10.1007/s10935-025-00829-y.

## Introduction

Adolescents spend a large amount of their time at school, which makes schools an important setting for promoting health (Marmot, 2003; Mielke & Farrington, 2021; Sawyer et al., 2021). Beyond the benefits of learning, school participation lays a foundation for healthy adolescent development and ideally provides students with the skills and competencies they need to successfully transition to adulthood (Heerde et al., 2023a; Jourdan et al., 2021). It is, therefore, not surprising that disruption to schooling, such as school suspensions, can have lasting negative consequences leading into young adulthood. Research shows that suspension during adolescence predicts homelessness in young adulthood, suggesting that it may be a point of intervention to reduce young adult homelessness (Heerde et al., 2021; van den Bree et al., 2009).

Suspension is one tool on a continuum of behavior management approaches used in school settings (Hemphill et al., 2017; Mielke & Farrington, 2021). Compared to moving students to different seating within the classroom or the use of detention as a short-term response, suspension is a more severe disciplinary approach designed to punish and then prevent behavior that is deemed unacceptable or that poses a risk to other students or staff by removing the student from the school environment (Camacho & Krezmien, 2020; Mielke & Farrington, 2021). There are multiple reasons for schools to suspend students, including misbehavior such as disruptive classroom behaviors to more challenging behaviors such as substance use and engagement in antisocial behavior or violence (e.g., Hemphill et al., 2020; Wilkerson & Afacan, 2022). Schools vary in their interpretation of student misbehavior; a behavior that is considered a minor indiscretion in one school may carry serious consequences in another. Schools also vary in their suspension policies and how strictly these policies are enforced in response to misbehavior or policy violations. There are also differences by country. For example, while suspension is used as a behavior management tool in both the United States and Australian school contexts, norms and policies around suspension that reflect a zero-tolerance approach to misbehavior are particularly common in the United States compared to Australia, where whole-school restorative approaches to addressing misbehavior are also common (Hemphill et al., 2016). Prior studies show rates of school suspension among adolescents in Victoria are lower than their counterparts in Washington State (Heerde et al., 2022a; Hemphill et al., 2020).

Past research has identified several risk factors associated with a higher likelihood of suspension. For example, using data from the International Youth Development Study (IYDS), Hemphill et al. (2014a) showed that alcohol use at the age of 13 increased the risk of suspension one year later. Additional risk factors for suspension across various adolescent social-ecological domains include male sex, lower socioeconomic status, behavioral difficulties (including engagement in antisocial or violent behaviors), academic underachievement, and lower commitment to schooling (e.g., Hemphill et al., 2014b, 2017; Sullivan et al., 2013).

There is also evidence of negative outcomes associated with suspension. Longitudinal studies demonstrate the negative effects suspension has on adolescent educational participation and outcomes (e.g., increased likelihood of future suspensions, school disengagement, reduced academic achievement, lower rates of school completion) and behavioral outcomes (e.g., future engagement in antisocial and violent behavior, substance use, contact with the justice system) (Arcia, 2006; Hemphill et al., 2016, 2017; Mielke & Farrington, 2021; Skiba & Rausch, 2013). For example, analysis of data from the IYDS showed that suspension uniquely predicted antisocial and violent behaviors and tobacco use, even after controlling for known risk factors such as prior antisocial behavior (Hemphill et al., 2006, 2009, 2012).

Young adults who experience homelessness commonly report disruption to school participation, including a suspension and expulsion, absenteeism, self-exclusionary behaviors (for example, truancy, engagement in behaviors likely to result in suspension or expulsion), and disconnection from school during adolescence (Heerde et al., 2020b; Robinson, 2021). As such, school policies, including those related to suspension, may constitute a point of intervention to reduce young adult homelessness at a population level. Despite its potential, research that examines this intervention approach is scant, and the existing literature is inconsistent. Using data from The National Longitudinal Study of Adolescent to Adult Health (Add Health), Shelton et al. (2009) found no effect of suspension or expulsion (retrospectively reported at age 18–26) on the risk for adult homelessness. Conversely, prior work using data from the IYDS showed that suspension between ages 13 to 15 was a strong predictor of homelessness in the past year by age 25 years (Odds Ratio 2.8) adjusting for a wide range of individual, family, peer, and community risk factors (Heerde et al., 2020a). Further, follow-up analyses mapping pathways to young adult homelessness showed that suspension at age 15 mediated associations between both family conflict and poor family management at age 13 and young adult homelessness (Heerde et al., 2021, 2022a).

Understanding the possible ways in which suspension during adolescence may be associated with homelessness in young adulthood is critically important. It is plausible that the exclusion of adolescents from school via suspension contributes to an increase in school disengagement, which is associated with school dropout, substance abuse, antisocial behavior, and justice system contact (Henry et al., 2012; Noltemeyer et al., 2015; Wolf & Kupchik, 2017), all commonly reported factors among young adults who experience homelessness (Heerde et al., 2023b). Further, removal from the school environment can disconnect adolescents from teachers and the protection that schools can provide in relation to facilitating access to support services (Heerde et al., 2023a; Mielke & Farrington, 2021). Identifying risk factors within school settings, such as student behaviors that may result in suspension, and understanding the enactment of policies that use suspension as a response to these behaviors, may help shape school-based prevention responses that seek to reduce risk and promote safe learning environments before more concerning health-compromising patterns (e.g., substance use, violence) or disengagement from school become established.

The present study used data from a population-based sample of young adult participants in the IYDS to examine the extent to which school behavior management policies and adolescent risk factors predict suspension by age 15 and young adult homelessness by age 25. We hypothesized that (1) both school-based behavior management policies and adolescent risk factors would be associated with young adult homelessness and (2) suspension would mediate the expected association between adolescent risk factors and young adult homelessness.

## Methods

### Sample

Data were drawn from a population-based sample of adolescent and young adult participants in the IYDS, a large, prospective, longitudinal study of the epidemiology of health and social behaviors in young people from Victoria, Australia, and Washington State, United States. Initially recruited as adolescents in state-representative secondary school samples, participants in both states have most recently been followed into young adulthood.

Details of the original IYDS sampling and recruitment methods have been previously published (McMorris et al., 2007). State-representative samples were achieved using a two-stage cluster sampling approach. In the first stage, public and private schools with Grades 5, 7, and 9 (younger, middle, and older cohorts, respectively) were randomly selected for recruitment using a probability proportionate to grade-level size sampling procedure (Kish, 1965). Three-hundred and sixty-eight schools in Washington State and 254 schools in Victoria were approached to participate. Of these, 155 in Washington State and 165 in Victoria agreed to participate (42% and 65%, respectively). In the second stage, one target classroom at each appropriate grade level, within each participating school, was randomly selected (McMorris et al., 2007). Across all grade levels, 73.5% of students in selected classrooms (n = 2884) in Victoria and 74.5% (n = 2885) in Washington State participated in the 2002 (baseline) survey. At the time of recruitment, Victoria and Washington State were similar in population size and urbanicity, had higher than national-average levels of educational participation, and had a low proportion of residents living in poverty (McMorris et al., 2007). To ensure seasonal and school year equivalence, annual surveys were conducted from February to June in Washington State and from May to November in Victoria.

**Fig. 1:**
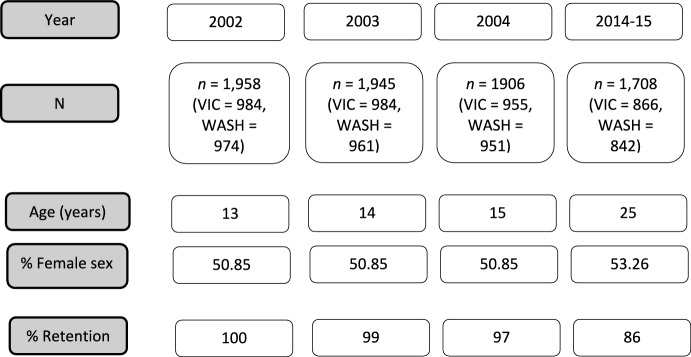
Sample recruitment and retention across the study waves. *Note*: VIC = Victoria. WASH = Washington State

This study analyzed data from the middle cohort (students in Grade 7 in 2002, n = 1,945 [984 in Victoria], 51% female; See Fig. 1) at ages 13 (median = 12.97 years), 14, 15, and 25 years (median = 25.11 years). Over 90% of the Victorian sample identified as Australian, and 65% of the Washington State sample identified as White. In Victoria, the remaining participants identified as 5% Asian, 1% Aboriginal and Torres Strait Islander, and < 1% African, Spanish/Hispanic/Latino, or Pacific Islander. In Washington, the remaining participants identified as 16% Hispanic/Latinx, 4% African American, 6% Native American, and 6% Asian/Pacific Islander. In young adulthood, participants self-reported their sexual identity, with a majority of the sample identifying as heterosexual (81% in Victoria, and 79% in Washington State).

### Procedure

In Australia, the University of Melbourne Human Ethics in Research Committee and the Royal Children's Hospital (Melbourne) approved the study. In the US, the University of Washington Human Subjects Institutional Review Board approved the study. Relevant educational authorities for public and private schools in both states provided permission to conduct the study in schools. Prior to study commencement, the IYDS design and methods underwent multiple processes to ensure cross-national validity and reduce method differences that are commonly seen in studies drawing international comparisons (Segall et al., 1998). These processes included matching sampling and recruitment strategies, surveys, and survey administration procedures. The survey also underwent cognitive pre-testing and piloting, including language review and cross-national item adaptation, to ensure content validity across the two states (McMorris et al., 2007). As the IYDS is a prospective, longitudinal study, it has detailed and comprehensive protocols for repeated follow-up of participants over time, designed to maximize participant retention.

In 2002, written parental consent and participant assent were obtained for all participants. Participant consent was obtained for the young adult survey. The adolescent surveys were administered to class groupings within schools, and the young adult survey was administered online. Surveys took 50–60 min to complete. During adolescence, Victorian participants received a small gift (e.g., stress ball), and Washington State participants received USD$10 at the completion of each survey. For the young adult survey, participants from both states were compensated with a USD/AUD$40 gift voucher.

At the time of the baseline survey, the study also included a school administrator survey completed by the school principal (or nominee) from each participating school. The survey was conducted via mail (97.4% participation rate; Evans-Whipp et al., 2015).

### Measures

The IYDS used self-report measures adapted from the Communities That Care youth survey (Arthur et al., 2002; Glaser et al., 2005). Survey measures were reviewed and adjusted to be developmentally appropriate as the sample aged from adolescence into young adulthood. Measures of school-level behavior management policies were specifically developed for the IYDS (Beyers et al., 2005; Evans‐Whipp et al., 2007). Survey measures analyzed in this study have demonstrated longitudinal validity and reliability in the state-based samples (Evans-Whipp et al., 2015; Heerde et al., 2021).

### Person-Level Variables

*Young adult homelessness (age 25)* was measured using the item “In the past year, have you been homeless (i.e., not had a regular place to live?)” Response options were dichotomous, ‘No’ (0, reference group) and ‘Yes’ (1).

*Suspension*. At ages 13, 14, and 15, students reported the number of times in the past year they had been suspended from school. Response options ranged from “never” (1) through to “40 + times” (8). At each age, responses were dichotomized (0 *not suspended*, 1 *suspended*). Scores were collapsed across ages to create a variable indexing suspension by age 15, “Not suspended by Age 15” (0, reference group) and “Suspended by Age 15” (1).

*Student-perceived likelihood of suspension/expulsion* (age 13). Participants were asked to indicate the severity of consequences for each of the following four scenarios: “If a student was found smoking cigarettes [drinking alcohol/ using marijuana/ using other illegal drugs] at school, which of the following would most likely happen?”. Proposed consequences were: ‘He or she would be talked to by a teacher about the dangers of (behavior),’ ‘He or she would be suspended,’ ‘He or she would be expelled,’ and ‘The police would be called.’ Response options for each of these consequences were ‘No’ (0) and ‘Yes’ (1). Dichotomized scores for each item were then collapsed to create a variable indexing likelihood of suspension/expulsion: “Not likely to be suspended/expelled” (0, reference group) and “Likely to be suspended/expelled” (1).

*Covariates (Ages 13 & 14)* included rebelliousness, non-violent antisocial behavior, violent antisocial behavior, and substance use. Three items were used to assess *rebelliousness* (e.g., “I ignore rules that get in my way”). Response options ranged from ‘definitely no’ (1) through to ‘definitely yes’ (4). Scores across each of these items were averaged to obtain a single scale score across ages 13 and 14. *Non-violent antisocial behavior* was measured using seven items, of which “How many times in the past year (12 months) have you stolen something worth more than $5/$10?” is an example. Participants reported their engagement in *violent antisocial behavior* using three items. “How many times in the past year have you beat up someone so badly that they probably needed to see a doctor or nurse?” was one of these items. Response options for the rebelliousness, non-violent, and violent antisocial behavior items ranged from ‘never’ (1) through to ‘40 + times’ (8). Within each scale (rebelliousness, non-violent, and violent antisocial behavior), item scores were averaged to obtain a single scale score across ages 13 and 14. Scores were coded such that higher scores indicated higher rebelliousness, non-violent, and violent antisocial behavior.

Participants self-reported their use of *alcohol, tobacco, and cannabis* by age 14 using the items: “In the past 30 days, on how many occasions (if any) have you had more than just a few sips of an alcoholic beverage (like beer, wine or liquor/spirits)? “, “How frequently have you smoked cigarettes in the past 30 days?” and “In the past 30 days on how many occasions (if any) have you: used marijuana (pot, weed, grass)?”. Response options for each item at ages 13 and 14 ranged from “never” (1) through to “40 + times” (8). Responses for each item were dichotomized: (“*no use”* [0, reference group], *used in the past 30 days* [1]) and collapsed to create use by age 14 measures of *alcohol, tobacco, and cannabis*.

*Demographic factors* Participants reported their *sex* (female [0], male [1]) and the *state* in which they resided (Washington State [0], Victoria [1]). *Family socioeconomic status* was created using parent (mother and father) self-reported annual family income (ranging from ‘less than $10,000’ to ‘$200,000 and above’) and the highest level of education (e.g., ‘less than secondary school,’ ‘completed secondary school,’ and ‘completed post-secondary school’), obtained in phone interviews conducted at the time of the 2002 survey.

### School-Level Variable

*School behavior management policy* was reported by school principals (or nominee) using three items: “When students are caught using or possessing tobacco on school grounds or at school events, how often are they …”, “When students are caught using, possessing or being under the influence of alcohol on school grounds or at school events, how often are they …”, and “When students are caught using, possessing, or being under the influence of illicit drugs on school grounds or at school events, how often are they…”. Response categories reflect the likelihood of specific consequences: ‘Referred to legal authorities (police),’ ‘Suspended from school,’ and ‘Expelled from school’ rated on a 4-point scale of ‘Almost or always (4)’, ‘Sometimes (3)’, ‘Rarely (2)’ and ‘Never (1)’. Scores for the three items were averaged to obtain a single scale score where higher scores indicated a greater likelihood of the consequence.

*Survey response accuracy* was examined at ages 13, 14, and 15. Responses were coded as questionable if participants reported (1) “I was not honest all of the time” when asked how honest they were when completing the survey, (2) use of a fictitious drug (included in the survey for accuracy checking) in the past month, and (3) drug use on > 120 occasions in the past month.

### Statistical Analysis

Descriptive analyses were conducted using Stata SE software for Windows, version 15.1 (StataCorp LLC, 2017). Tests of differences in means and frequencies for adolescent predictors and young adult homelessness were completed using *t-*tests and chi-square analyses, respectively. Effect sizes were calculated using pooled standard deviations (Cohen, 1977). Zero-order correlations were examined to identify highly correlated pairs or sets of variables that might have resulted in collinearity in the subsequent path modeling (Tabachnick & Fidell, 2013). Fifteen participants at age 13, 35 at age 14, and 27 at age 15 met the criteria for questionable responses (e.g., use of a fictitious drug) and were excluded from the analyses. The percentage of missing data on the analyzed variables ranged from 0.05% to 12.60% (*M* = 1.47%).

We estimated two multilevel, longitudinal path models using Mplus, version 8.7 (Muthén & Muthén, 2017). Full information maximum likelihood estimation was used to minimize potential bias due to missing data using the robust Maximum Likelihood Estimator and the weighted least squares means and variance adjusted estimator (Muthén & Muthén, 2017; Schafer & Graham, 2002). We started with estimating the person-level portion of the model. Indirect (i.e., mediated) effects of individual predictors on age 25 homelessness were estimated using the *model indirect* function. We then included the school-level portion of the model by clustering by school and incorporating school principal (or nominee) reports of school-level behavior management policies. Demographic factors were included in all analyses. Model fit indices were examined in accordance with current recommendations (Cangur & Ercan, 2015). Standardized parameter estimates are presented.

## Results

Young adults in Washington reported a significantly higher rate of homelessness than in Victoria (Table 1), with rates of 5.25% [95% CI 3.92, 6.97] and 3.25% [95% CI = 2.25, 4.67], respectively. They also reported significantly higher family socio-economic status and higher rates of both marijuana use by age 14 and suspension by age 15 compared to Victorian respondents. By age 14, Victorian participants showed higher levels of rebelliousness and rates of alcohol and tobacco use compared to those in Washington State. They also reported a greater perceived likelihood of suspension/expulsion being enacted by their school. Intercorrelations were low-moderate and in the expected direction (range *r* =  < 0.01 to *r* = 0.77; see Table S1, Online Resource 1). Overall, young adults who reported experiencing homelessness at age 25 reported higher levels of adolescent rebelliousness and higher rates of non-violent and violent behavior, tobacco and cannabis use, and suspension compared to those who had not experienced homelessness (Table S2, Online Resource 1). Notably, over half of young adults who reported past year homelessness at age 25 reported a history of suspension in adolescence.

**Table 1:** Summary statistics and tests of differences for individual and school predictors and young adult homelessness, by state

	Combined sample (n = 1945)		Victoria (n = 984)		Washington state (n = 961)		*p* value	Difference *t*/χ^2^	Effect size *d*
	%		%		%				
Young adult homelessness	4.24		3.25		5.25		.040	4.200	.099
*Demographic factors*									
Female sex	49.15		49.19		49.12		.975	.001	-.001
	M(SD)	95% CI	M(SD)	95% CI	M(SD)	95% CI			
Higher family socio-economic status	2.00(.43)	1.98, 2.02	1.94(.49)	1.91, 1.97	2.06(.37)	2.03, 2.08	< .001	5.809	.268
*Individual predictors*									
Age 13–14 Rebelliousness	1.87(.58)	1.84, 1.89	1.90(.59)	1.87, 1.94	1.83(.56)	1.79, 1.86	.003	-2.933	-.133
Age 13–14 Non-violent antisocial behavior	1.11(.33)	1.10, 1.13	1.11(.37)	1.09, 1.13	1.12(.29)	1.10, 1.14	.457	.743	.033
Age 13–14 Violent antisocial behavior	1.09(.34)	1.08, 1.11	1.10(.38)	1.08, 1.13	1.08(.29)	1.06, 1.09	.069	-1.820	-.083
	%		%		%				
Alcohol use by age 14	39.90		52.54		26.95		< .001	132.766	-.541
Tobacco use by age 14	13.79		16.48		11.03		< .001	12.143	-.158
Marijuana use by age 14	8.89		6.20		11.65		< .001	17.855	.192
*School predictors*									
Suspension by age 15	23.39		21.24		25.60		.023	5.154	.103
Student-perceived likelihood of suspension/expulsion	95.17		96.14		94.17		.043	4.804	-.092
School-level behavior management policy	2.28(.63)	2.25, 2.31	2.61(.61)	2.57, 2.65	1.95(.43)	1.92, 1.97	< .001	-27.354	-1.260

*M* = mean, *SD* = standard deviation. % = per cent. χ^2^ = chi-square. *t* = *t-*statistic*.* 95% CI = 95% confidence interval. Young adult homelessness (past year; coded 0 = no, 1 = yes). School suspension by age 15 (coded 0 = no, 1 = yes). Alcohol, tobacco and marijuana use (coded 0 = no use, 1 = past month use). Student-perceived likelihood of suspension/expulsion (coded 0 = Not likely to be suspended/expelled, 1 = Likely to be suspended/expelled). Statistically significant state differences for continuous variables calculated using independent *t*-tests. Statistically significant state differences for dichotomous variables calculated using chi-square tests

**Fig. 2:**
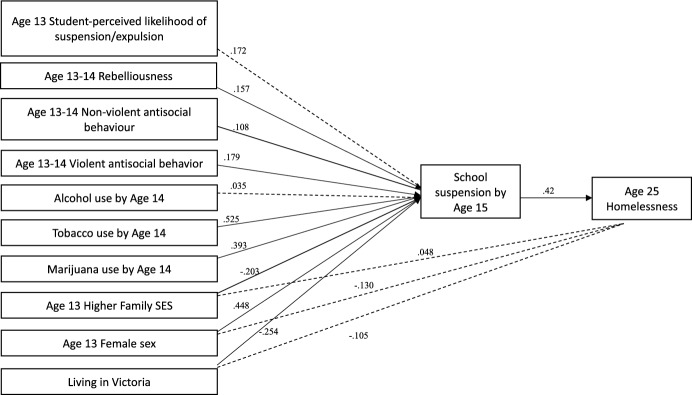
Results from the path model: individual and school predictors of young adult homelessness. *Note*. Model fit statistics: χ^2^(21, n = 1879) = 235.23, *p* < .0001, CFI = .987, TLI = 0.962, RMSEA estimate = 0.014. Standardized parameter estimates shown. Dashes indicate non-significant paths. Solid lines indicate paths significant at *p* < .05. Victoria (coded 0 = Washington State, 1 = Victoria). Alcohol, tobacco and marijuana use (coded 0 = no use, 1 = past month use). School suspension by age 15 (coded 0 = no, 1 = yes). Young adult homelessness (past year; coded 0 = no, 1 = yes). Student-perceived likelihood of suspension/expulsion (coded 0 = Not likely to be suspended/expelled, 1 = Likely to be suspended/expelled)

Path model analyses of person-level data showed that early adolescent rebelliousness, violent and non-violent antisocial behavior, and tobacco or marijuana use predicted suspension by age 15 (see Fig. 2). Male sex, lower family socioeconomic status, and living in Washington State also predicted suspension. Student perceptions of the likelihood of suspension/expulsion were unrelated to suspension. Suspension uniquely increased the risk of young adult homelessness. No other model variables were uniquely associated with homelessness. However, statistically significant indirect effects of rebelliousness, violent antisocial behavior, tobacco use, and marijuana use on young adult homelessness were found. Full results for the model are included in Table S3 (Online Resource 1).

**Fig. 3:**
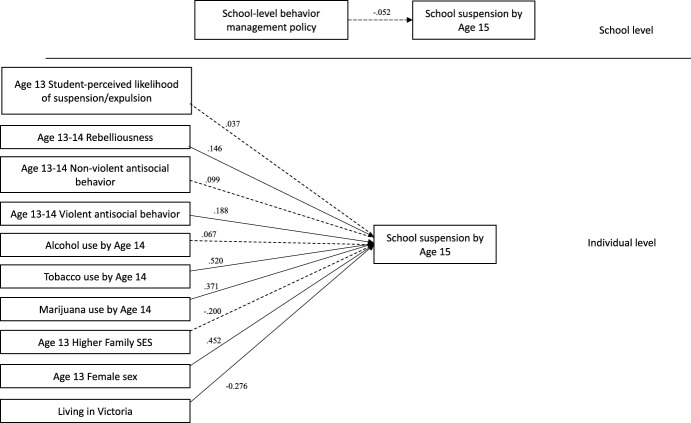
Results from the multilevel model: individual predictors of school suspension. *Note*. The model is fully saturated. Standardized parameter estimates shown. Dashes indicate non-significant paths. Solid lines indicate paths significant at *p* < .05. Victoria (coded 0 = Washington State, 1 = Victoria). Alcohol, tobacco and marijuana use (coded 0 = no use, 1 = past month use). School suspension by Age 15 (coded 0 = no, 1 = yes). Student-perceived likelihood of suspension/expulsion (coded 0 = Not likely to be suspended/expelled, 1 = Likely to be suspended/expelled)

Given the small proportion of individuals who experienced homelessness, adding school principal reports of behavior management policy (school-level) and accounting for school clustering resulted in problems with model estimation. As current and prior analyses confirmed an association between suspension and later homelessness, we dropped homelessness from between-person models and focused on predicting suspension as a more prevalent outcome. The between-person school behavior management policy variable was not uniquely related to suspension (Fig. 3). Instead, suspension was predicted by within-person risk behaviors, male sex, and living in Washington State. Full results for the model are included in Table S4 (Online Resource 1).

## Discussion

The current study tested whether adolescent problem behaviors, student-perceived school behavior management policies, and school-level behavior management policies were related to suspension by age 15 and, in turn, the probability of homelessness at age 25. Results showed that suspension at age 15 predicted young adult homelessness at the person-level. Interestingly, after controlling for individual factors, school behavior management policy, defined as the likelihood of suspension/expulsion for substance use perceived by students or reported by principals, was not related to a history of suspension at either the individual or school levels. Rather, the individual factors that included adolescent rebelliousness, non-violent and violent antisocial behavior, and substance use predicted suspension. Further, these individual level problem behaviors were indirectly related to increased risk for homelessness at age 25. This finding indicates that suspension is a risk factor for later homelessness and suggests that prevention programs targeting problem behaviors and strengthening bonds to school could be effective strategies for reducing young adult homelessness (Markowitz, 2017; Mielke & Farrington, 2021).

In the current sample, more than one in two (55.56%) young adults who experienced homelessness within the past year at the age of 25 reported being suspended from school during adolescence. High rates of suspension among this group are worrying, particularly in the context of existing research that links suspension with various adverse outcomes in young adulthood (Arcia, 2006; Hemphill et al., 2016; Mielke & Farrington, 2021; Skiba & Rausch, 2013). These findings are also important given that school connectedness has been shown to enhance student mental health (Jourdan et al., 2021; Raniti et al., 2022) and that many young adults who experience homelessness report barriers to schooling which often lead to school disengagement, exclusion, and early school leaving (Heerde et al., 2020b; Robinson, 2021). Our findings add to the evidence by identifying severe, long-term negative consequences of suspension, and suggest a critical need for effective discipline approaches that do not involve disconnecting adolescents from their school environment.

Although the intersection of housing and education as determinants of health equity has been recognized (Lancet Public Health, 2020; Marmot & Allen, 2014), the specific risk factors that define pathways to homelessness across the life course are not well understood. Similarly, school-based responses to homelessness have been historically neglected. Current strategies are overwhelmingly designed to reduce early school leaving among adolescents at risk of homelessness (e.g., MacKenzie, 2016; Malenfant et al., 2020). However, the evidence supporting the effectiveness of early intervention responses in reducing rates of homelessness among adolescents or improving long-term educational or housing outcomes is limited (Morton et al., 2020; Toumbourou & Heerde, 2022). In this context, responses to young adult homelessness may be strengthened by using whole-school approaches as these encompass the spectrum of responses from prevention through to postvention support (Heerde et al., 2023a). Frameworks such as health-promoting schools (Langford et al., 2015; World Health Organization, 2021), comprehensive school health programs (American School Health Association, 1995), and the Whole School, Whole Community, Whole Child model (Centers for Disease Control & Prevention, 2023) are designed to facilitate positive education and health outcomes through a whole-school approach. Yet there are important evidence gaps that could inform the application of these frameworks within specific contexts, including risk factors that define pathways to homelessness in the transition from adolescence to adulthood.

The finding that suspension was predicted by substance use and violent antisocial behavior underscores the need for preventive intervention in multiple areas of adolescents’ lives and hints that such interventions may also help to prevent later homelessness (Bonell et al., 2018). These behaviors might not only influence adolescents’ behavioral and emotional disengagement from schooling but also instigate the enforcement of exclusionary school-based behavior management policies (e.g., suspension, expulsion). Moreover, these behaviors may share common antecedents (e.g., low family SES, family conflict; Heerde et al., 2022a; Hemphill et al., 2015). Young adults who experience homeless often experience health issues stemming from their marginalization, including poorer physical and mental health (Edidin et al., 2012; Hodgson et al., 2013), high rates of substance use (Kulik et al., 2011), and high rates of preventable morbidities arising from violence (Boivin et al., 2005). Widespread implementation of effective substance use and positive youth development programs, such as those listed by Blueprints for Healthy Youth Development (blueprintsprograms.org) that have been shown to reduce adolescent substance use and antisocial behavior and increase effective school behavior management practices (e.g., Communities That Care; Hawkins et al., 2008), may help to prevent later homelessness and associated poor health by reducing rates of suspensions.

We have previously shown that there are higher rates of homelessness in Washington State than in Victoria, Australia (Heerde et al., 2021, 2022a). In this study, we show that Washington State also has higher rates of suspension than Victoria. Differences in cultural and economic contexts, such as funding for social safety nets and housing investments between the two states, as well as differences in school behavior management policy orientations (a harm minimization approach in Victoria compared to a zero-tolerance approach in Washington State), likely underpin these findings. Future analyses should investigate these differences to identify the features of state and country policy that influence these housing and educational outcomes.

### Study Strengths and Limitations

A significant strength of this study is the analysis of longitudinal data collected from a representative population-based sample that examined various predictors of homelessness that were measured across the formative years of early adolescence through to young adulthood. Data were collected from the cross-national samples using identical methods of recruitment, survey, and longitudinal follow-up (McMorris et al., 2007). Study retention rates remain high.

We acknowledge several study limitations. The single-item measure of homelessness, although typical in studies of adolescents and young adults (Heerde & Hemphill, 2016), limited our capacity to examine the variety of housing circumstances that represent homelessness such as couch surfing or staying with friends temporarily (e.g., couch surfing, staying with friends temporarily; Amore et al., 2011). However, prior qualitative follow-up of study participants demonstrated this single-item measure reflected the various forms of homelessness experienced by young adults (e.g. rough sleeping, couch surfing; Heerde et al., 2020b). Homelessness that may have occurred prior to age 25 was not assessed. While an important proportion, the relatively small number of participants experiencing homelessness limited our ability to test the direct association between school-level measures of policy and homelessness. A further limitation of this study is that is that the measure of school suspension did not distinguish between in-school suspension (removal of a student from the classroom environment where the student is still required to attend school) or out-of-school suspension (removal of a student from the physical school environment where the student is not permitted to attend school). Whether in- and out-of-school suspension are associated with later homelessness in similar ways requires additional investigation. The measure of school-level behavior management policy assumes that the policies are consistently enacted, which may not be the case. Although considered reliable in studies of adolescents and young adults (Jolliffe et al., 2003), the measures analyzed were based on self-report data. Participants were not asked about their gender identity in adolescence beyond the binary question of male or female, which was consistent with approaches at the time of the survey. High proportions of participants identifying as heterosexual and White (in Washington State) and Australian (in Victoria) means our analyses did not control for or examine differences in associations by sexual identity, gender identity, race, or ethnicity. Therefore, our findings are generalizable only to the state and cohort samples analyzed. We note that most population-based cohort studies analyzing risk for homelessness are commonly limited by a low prevalence of both homelessness and proportions of participants with diverse gender, sexuality, ethnicity and racial identities, meaning that there is a lack of statistical power to examine potential relationships between these determinants and later homelessness (Heerde et al., 2024; Shelton et al., 2009; van den Bree et al., 2009). Future research could consider the use of multi-cohort life course approaches that bring together and harmonize data across several high-quality cohort studies as a strategy to overcome this (Greenwood et al., 2024; Heerde et al., 2022b; O’Connor et al., 2022).

## Conclusions

Young adults experiencing homelessness are identifiable in population-based samples. This study identified multiple, malleable adolescent risk factors for young adult homelessness, including substance use, antisocial behavior, rebelliousness, and suspension. Suspension emerged as a critical mediating variable for later homelessness. Widespread implementation of effective prevention programming is recommended to reduce the rate of adolescent substance use and antisocial behavior and reduce suspension, with a wider goal of shifting the rate of young adult homelessness.

## Supplementary Information

Below is the link to the electronic supplementary material.

Supplementary file1 (DOCX 36 KB)

## Data Availability

The datasets analysed during the current study are not publicly available but may be available from the corresponding author on reasonable request via the Melbourne Children’s LifeCourse Initiative (https://lifecourse.melbournechildrens.com/).

## References

[CR1] American School Health Association. (1995). *Guidelines for comprehensive school health programs*. American School Health Association.

[CR2] Amore, K., Baker, M., & Howden-Chapman, P. (2011). The ETHOS definition and classification of homelessness: An analysis. *European Journal of Homelessness,**5*(2), 19–37.

[CR3] Arcia, E. (2006). Achievement and enrollment status of suspended students: Outcomes in a large, multicultural school district. *Education and Urban Society,**38*(3), 359–369.

[CR4] Arthur, M. W., Hawkins, J. D., Pollard, J. A., Catalano, R. F., & Baglioni, A., Jr. (2002). Measuring risk and protective factors for use, delinquency, and other adolescent problem behaviors: The communities that care youth survey. *Evaluation Review,**26*(6), 575–601. 12465571 10.1177/0193841X0202600601

[CR5] Beyers, J. M., Evans-Whipp, T., Mathers, M., Toumbourou, J. W., & Catalano, R. F. (2005). A cross-national comparison of school drug policies in Washington State, United States, and Victoria, Australia. *Journal of School Health,**75*(4), 134–140. 15987007 10.1111/j.1746-1561.2005.00011.x

[CR6] Boivin, J.-F., Roy, É., Haley, N., & Du Fort, G. G. (2005). The health of street youth: A Canadian perspective. *Canadian Journal of Public Health,**96*, 432–437. 16350867 10.1007/BF03405183 PMC6975913

[CR7] Bonell, C., Allen, E., Warren, E., McGowan, J., Bevilacqua, L., Jamal, F., Legood, R., Wiggins, M., Opondo, C., & Mathiot, A. (2018). Effects of the learning together intervention on bullying and aggression in English secondary schools (INCLUSIVE): A cluster randomised controlled trial. *The Lancet,**392*(10163), 2452–2464. 10.1016/S0140-6736(18)31782-3 PMC6286420 30473366

[CR8] Camacho, K. A., & Krezmien, M. P. (2020). A statewide analysis of school discipline policies and suspension practices. *Preventing School Failure: Alternative Education for Children and Youth,**64*(1), 55–66.

[CR9] Cangur, S., & Ercan, I. (2015). Comparison of model fit indices used in structural equation modeling under multivariate normality. *Journal of Modern Applied Statistical Methods,**14*(1), 14.

[CR10] Centers for Disease Control and Prevention. (2023). *Whole School, Whole Communty, Whole Child (WSCC)*. Centers for Disease Control and Prevention,. Retrieved 07/08/2023 from https://www.cdc.gov/healthyschools/wscc/index.htm#:~:text=The%20WSCC%20model%20is%20student,Physical%20education%20and%20physical%20activity

[CR11] Cohen, J. (1977). *Statistical power analysis for the behavioural sciences (Revised edition)*. Academic Press.

[CR12] Edidin, J. P., Ganim, Z., Hunter, S. J., & Karnik, N. S. (2012). The mental and physical health of homeless youth: A literature review. *Child Psychiatry & Human Development,**43*, 354–375. 22120422 10.1007/s10578-011-0270-1

[CR13] Evans-Whipp, T. J., Bond, L., Toumbourou, J. W., & Catalano, R. F. (2007). School, parent, and student perspectives of school drug policies. *Journal of School Health,**77*(3), 138–146. 17302856 10.1111/j.1746-1561.2007.00183.x

[CR14] Evans-Whipp, T. J., Plenty, S. M., Catalano, R. F., Herrenkohl, T. I., & Toumbourou, J. W. (2015). Longitudinal effects of school drug policies on student marijuana use in Washington State and Victoria, Australia. *American Journal of Public Health,**105*(5), 994–1000. 25790384 10.2105/AJPH.2014.302421 PMC4386529

[CR15] Glaser, R. R., Horn, M. L. V., Arthur, M. W., Hawkins, J. D., & Catalano, R. F. (2005). Measurement properties of the Communities That Care® Youth Survey across demographic groups. *Journal of Quantitative Criminology,**21*(1), 73–102.

[CR16] Greenwood, C. J., Letcher, P., Laurance, E., Boden, J. M., Foulds, J., Spry, E. A., Kerr, J. A., Toumbourou, J. W., Heerde, J. A., & Nolan, C. (2024). The monitoring illicit substance use consortium: A study protocol. *JAACAP Open,**2*, 311–322. 39697393 10.1016/j.jaacop.2024.03.002 PMC11650658

[CR17] Hawkins, J. D., Brown, E. C., Oesterle, S., Arthur, M. W., Abbott, R. D., & Catalano, R. F. (2008). Early effects of communities that care on targeted risks and initiation of delinquent behavior and substance use. *Journal of Adolescent Health,**43*(1), 15–22. 10.1016/j.jadohealth.2008.01.022 PMC3867289 18565433

[CR18] Heerde, J., Raniti, M., & Sawyer, S. (2023a). Health promoting schools: An opportunity to strengthen responses to homelessness and health disparities in children and adolescents. Parity, Accepted 16 March 2023.

[CR19] Heerde, J., Bailey, J., Kelly, A., McMorris, B. J., Patton, G. C., & Toumbourou, J. W. (2021). Life-course predictors of homelessness from adolescence into adulthood: A population-based cohort study. *Journal of Adolescence,**91*, 15–24. 10.1016/j.adolescence.2021.06.007 34271292 10.1016/j.adolescence.2021.06.007 PMC8423126

[CR20] Heerde, J. A., Bailey, J. A., McMorris, B. J., Patton, G. C., Sawyer, S. M., & Toumbourou, J. W. (2024). Predictors of housing insecurity in young adulthood. *Emerging Adulthood,**12*, 607–619. 10.1177/21676968241253878 PMC12826551 41585472

[CR21] Heerde, J., Bailey, J., Toumbourou, J. W., Rowland, B., & Catalano, R. F. (2020a). Longitudinal associations between early-mid adolescent risk and protective factors and young adult homelessness in Australia and the United States. *Prevention Science,**21*(4), 557–567. 10.1007/s11121-020-01092-9 31965426 10.1007/s11121-020-01092-9 PMC7166182

[CR22] Heerde, J., Bailey, J., Toumbourou, J. W., Rowland, B., & Catalano, R. F. (2022a). Adolescent antecedents of young adult homelessness: A cross-national path analysis. *Prevention Science,**23*(1), 85–95. 10.1007/s11121-021-01267-y 34181152 10.1007/s11121-021-01267-y PMC8712626

[CR23] Heerde, J. A., Begun, S., Pearce, L., Kacholia, V., Logie, C., Patton, G. C., & Sawyer, S. (2023b). Homelessness and housing insecurity. In E. Neblett & W. Troop-Gordon (Eds.), *Encyclopedia of adolescence* (2nd ed.). Elsevier.

[CR24] Heerde, J. A., & Hemphill, S. A. (2016). Sexual risk behaviors, sexual offenses, and sexual victimization among homeless youth: A systematic review of associations with substance use. *Trauma, Violence, & Abuse,**17*(5), 468–489. 10.1177/1524838015584371 10.1177/1524838015584371 25985990

[CR25] Heerde, J., Olsson, C. A., Sawyer, S., & Patton, G. C. (2022b). Strategic directions for preventing youth homelessness in Australia: A new role for research evidence. *Parity,**35*(8), 43–45.

[CR26] Heerde, J. A., Pallotta-Chiarolli, M., & Parolini, A. (2020b). “I dropped out early”: School Disengagement and Exclusion Among Young People Experiencing Homelessness. In P. Towl & S. Hemphill (Eds.), *Safe, Supportive, and Inclusive Learning Environments for Young People in Crisis and Trauma: Plaiting the Rope. *Routledge. 10.4324/9780429282102

[CR27] Hemphill, S., Broderick, D., & Heerde, J. (2017). Positive associations between school suspension and student problem behaviour: A summary of recent Australian findings. *Trends and Issues in Crime and Criminal Justice,**531*, 1–3.

[CR28] Hemphill, S. A., Heerde, J. A., Herrenkohl, T. I., & Farrington, D. P. (2015). Within-individual versus between-individual predictors of antisocial behaviour: A longitudinal study of young people in Victoria, Australia. *Australian & New Zealand Journal of Criminology,**48*(3), 429–445. 10.1177/0004865815589829 28123186 10.1177/0004865815589829 PMC5257254

[CR29] Hemphill, S. A., Heerde, J. A., Herrenkohl, T. I., Toumbourou, J. W., & Catalano, R. F. (2012). The impact of school suspension on student tobacco use: A longitudinal study in Victoria, Australia, and Washington State. *United States. Health Education & Behavior,**39*(1), 45–56. 21586667 10.1177/1090198111406724 PMC3158957

[CR30] Hemphill, S., Heerde, J. A., & McMorris, B. J. (2016). Is internal suspension associated with better student outcomes than external suspension? In P. Towl & S. Hemphill (Eds.), *Locked out: school exclusion contexts in Australia and Aotearoa New Zealand* (p. 47). New Zealand Council for Educational Research.

[CR31] Hemphill, S. A., Heerde, J. A., Scholes-Balog, K. E., Herrenkohl, T. I., Toumbourou, J. W., & Catalano, R. F. (2014a). Effects of early adolescent alcohol use on mid-adolescent school performance and connection: A longitudinal study of students in Victoria, Australia and Washington State, United States. *Journal of School Health,**84*(11), 706–715. 10.1111/josh.12201 25274170 10.1111/josh.12201 PMC4196706

[CR32] Hemphill, S. A., Plenty, S. M., Bond, L., Herrenkohl, T. I., Toumbourou, J. W., & Catalano, R. F. (2020). Demographic and socioeconomic predictors of school suspension: A longitudinal study in Victoria, Australia, and Washington State, United States. *Safe, Supportive, and inclusive learning environments for young people in crisis and trauma* (pp. 27–39). Routledge.

[CR33] Hemphill, S. A., Plenty, S. M., Herrenkohl, T. I., Toumbourou, J. W., & Catalano, R. F. (2014b). Student and school factors associated with school suspension: A multilevel analysis of students in Victoria, Australia and Washington State, United States. *Children and Youth Services Review,**36*, 187–194. 24860205 10.1016/j.childyouth.2013.11.022 PMC4028069

[CR34] Hemphill, S. A., Smith, R., Toumbourou, J. W., Herrenkohl, T. I., Catalano, R. F., McMorris, B. J., & Romanuik, H. (2009). Modifiable determinants of youth violence in Australia and the United States: A longitudinal study. *Australian & New Zealand Journal of Criminology,**42*(3), 289–309. 20204170 10.1375/acri.42.3.289 PMC2830014

[CR35] Hemphill, S. A., Toumbourou, J. W., Herrenkohl, T. I., McMorris, B. J., & Catalano, R. F. (2006). The effect of school suspensions and arrests on subsequent adolescent antisocial behavior in Australia and the United States. *Journal of Adolescent Health,**39*(5), 736–744. 10.1016/j.jadohealth.2006.05.010 17046511

[CR36] Henry, K. L., Knight, K. E., & Thornberry, T. P. (2012). School disengagement as a predictor of dropout, delinquency, and problem substance use during adolescence and early adulthood. *Journal of Youth and Adolescence,**41*, 156–166. 21523389 10.1007/s10964-011-9665-3 PMC4516271

[CR37] Hodgson, K. J., Shelton, K. H., van den Bree, M. B., & Los, F. J. (2013). Psychopathology in young people experiencing homelessness: A systematic review. *American Journal of Public Health,**103*(6), e24–e37. 23597340 10.2105/AJPH.2013.301318 PMC3698723

[CR38] Jolliffe, D., Farrington, D. P., Hawkins, J. D., Catalano, R. F., Hill, K. G., & Kosterman, R. (2003). Predictive, concurrent, prospective and retrospective validity of self-reported delinquency. *Criminal Behaviour and Mental Health,**13*(3), 179–197. 14654870 10.1002/cbm.541

[CR39] Jourdan, D., Gray, N. J., Barry, M. M., Caffe, S., Cornu, C., Diagne, F., El Hage, F., Farmer, M. Y., Slade, S., & Marmot, M. (2021). Supporting every school to become a foundation for healthy lives. *The Lancet Child & Adolescent Health,**5*(4), 295–303. 33485407 10.1016/S2352-4642(20)30316-3

[CR40] Kish, L. (1965). *Survey sampling*. Wiley.

[CR41] Kulik, D. M., Gaetz, S., Crowe, C., & Ford-Jones, E. (2011). Homeless youth’s overwhelming health burden: A review of the literature. *Paediatrics & Child Health,**16*(6), e43–e47. 22654549 10.1093/pch/16.6.e43 PMC3328221

[CR42] Lancet Public Health. (2020). Education: A neglected social determinant of health. *The Lancet. Public Health,**5*(7), e361. 32619534 10.1016/S2468-2667(20)30144-4 PMC7326385

[CR43] Langford, R., Bonell, C., Jones, H., Pouliou, T., Murphy, S., Waters, E., Komro, K., Gibbs, L., Magnus, D., & Campbell, R. (2015). The world health organization’s health promoting schools framework: A cochrane systematic review and meta-analysis. *BMC Public Health,**15*, 1–15. 25886385 10.1186/s12889-015-1360-y PMC4339015

[CR44] MacKenzie, D. (2016). COSS: Building a’community of schools and services’ model. *Parity,**29*(6), 19–21.

[CR45] Malenfant, J., Schwan, K., French, D., Gaetz, S., & Redman, M. (2020). *Preventing youth homelessness in the Canadian education system: Young people speak out*. Canadian Observatory on Homelessness Press.

[CR46] Markowitz, A. J. (2017). Associations between school connection and depressive symptoms from adolescence through early adulthood: Moderation by early adversity. *Journal of Research on Adolescence,**27*(2), 298–311. 28876520 10.1111/jora.12275

[CR47] Marmot, M. G. (2003). Understanding social inequalities in health. *Perspectives in Biology and Medicine,**46*(3), S9–S23. 14563071

[CR48] Marmot, M., & Allen, J. J. (2014). *Social determinants of health equity* (Vol. 104, pp. S517–S519). American Public Health Association. 10.2105/AJPH.2014.302200 PMC4151898 25100411

[CR49] McMorris, B., Hemphill, S., Toumbourou, J., Catalano, R., & Patton, G. (2007). Prevalence of substance use and delinquent behavior in adolescents from Victoria, Australia and Washington State United States. *Health Education & Behavior,**34*(4), 634–650. 16740513 10.1177/1090198106286272

[CR50] Mielke, M., & Farrington, D. P. (2021). School-based interventions to reduce suspension and arrest: A meta-analysis. *Aggression and Violent Behavior,**56*, 101518.

[CR51] Morton, M. H., Kugley, S., Epstein, R., & Farrell, A. (2020). Interventions for youth homelessness: A systematic review of effectiveness studies. *Children and Youth Services Review,**116*, 105096.

[CR52] Muthén, L. K., & Muthén, B. O. (2017). *Mplus Version 8 user’s guide*. Muthén & Muthén.

[CR53] Noltemeyer, A. L., Ward, R. M., & Mcloughlin, C. (2015). Relationship between school suspension and student outcomes: A meta-analysis. *School Psychology Review,**44*(2), 224–240.

[CR54] O’Connor, M., Spry, E., Patton, G., Moreno-Betancur, M., Arnup, S., Downes, M., Goldfeld, S., Burgner, D., & Olsson, C. A. (2022). Better together: Advancing life course research through multi-cohort analytic approaches. *Advances in Life Course Research,**53*, 100499. 36652217 10.1016/j.alcr.2022.100499

[CR55] Raniti, M., Rakesh, D., Patton, G. C., & Sawyer, S. M. (2022). The role of school connectedness in the prevention of youth depression and anxiety: A systematic review with youth consultation. *BMC Public Health,**22*(1), 2152. 36424575 10.1186/s12889-022-14364-6 PMC9694921

[CR56] Robinson, C. (2021). Reforming the engagement of schools with unaccompanied homeless children. *Oxford Research Encyclopedia of Education. *UK: Oxford University Press.

[CR57] Sawyer, S. M., Raniti, M., & Aston, R. (2021). Making every school a health-promoting school. *The Lancet Child & Adolescent Health,**5*(8), 539–540. 34181886 10.1016/S2352-4642(21)00190-5 PMC9765438

[CR58] Schafer, J. L., & Graham, J. W. (2002). Missing data: Our view of the state of the art. *Psychological Methods,**7*(2), 147–177. 12090408

[CR59] Segall, M., Lonner, W., & Berry, J. (1998). Cross-cultural psychology as a scholarly discipline: On the flowering of culture in behavioral research. *American Psychologist,**53*(10), 1101.

[CR60] Shelton, K. H., Taylor, P. J., Bonner, A., & van den Bree, M. (2009). Risk factors for homelessness: Evidence from a population-based study. *Psychiatric Services,**60*(4), 465–472. 19339321 10.1176/ps.2009.60.4.465

[CR61] Skiba, R. J., & Rausch, M. K. (2013). Zero tolerance, suspension, and expulsion: Questions of equity and effectiveness. *Handbook of classroom management* (pp. 1073–1100). UK: Routledge.

[CR62] StataCorp LLC. (2017). *Stata: Statistics/data analysis*. In (Version 15:1 SE edition) StataCorp LLC.

[CR63] Sullivan, A. L., Klingbeil, D. A., & Van Norman, E. R. (2013). Beyond behavior: Multilevel analysis of the influence of sociodemographics and school characteristics on students’ risk of suspension. *School Psychology Review,**42*(1), 99–114.

[CR64] Tabachnick, B. G., & Fidell, L. S. (2013). *Using multivariate statistics*. Allyn and Bacon.

[CR65] Toumbourou, J. W., & Heerde, J. A. (2022). Evidence on programs to address youth homelessness: An evidence check rapid review. *Parity,**35*, 43–45.

[CR66] van den Bree, M. B., Shelton, K., Bonner, A., Moss, S., Thomas, H., & Taylor, P. J. (2009). A longitudinal population-based study of factors in adolescence predicting homelessness in young adulthood. *Journal of Adolescent Health,**45*(6), 571–578. 10.1016/j.jadohealth.2009.03.027 19931829

[CR67] Wilkerson, K. L., & Afacan, K. (2022). Repeated school suspensions: Who receives them, what reasons are given, and how students fare. *Education and Urban Society,**54*(3), 249–267.

[CR68] Wolf, K. C., & Kupchik, A. (2017). School suspensions and adverse experiences in adulthood. *Justice Quarterly,**34*(3), 407–430.

[CR69] World Health Organization. (2021). *Making every school a health-promoting school–Global standards and indicators* [Strategy]. https://www.who.int/publications/i/item/9789240025059

