# Supplementary material for: School Suspension as a Predictor of Young Adult Homelessness: The International Youth Development Study
Source: J Prev (2022). 2025 Feb 16;46(4):467–85. doi: 10.1007/s10935-025-00829-y (PMC12289435; doi:10.1007/s10935-025-00829-y)
Supplement: Supplementary file 1 — Supplementary file1 (DOCX 36 KB) [file 10935_2025_829_MOESM1_ESM.docx]

**School suspension as a predictor of young adult homelessness: the International Youth Development Study.**

Jessica A Heerde, PhD^1,2,3,4,5^; Jennifer A Bailey, PhD^6^; Gabriel J. Merrin^7^; Monika Raniti, PhD^1,4^; George C. Patton^1,3,4^; John W. Toumbourou, PhD^8^; Susan M. Sawyer^1,3,4^

^1^Department of Paediatrics, The University of Melbourne, Australia

^2^Department of Social Work, The University of Melbourne, Australia.

^3^Centre for Adolescent Health, Royal Children's Hospital, Australia

^4^Murdoch Children’s Research Institute, Australia

^5^School of Population Health, Curtin University, Australia

^6^Social Development Research Group, School of Social Work, University of Washington, USA

^7^Department of Human Development and Family Science, Syracuse University, USA

^8^Centre for Social and Early Emotional Development, School of Psychology, Deakin University, Australia.

*Corresponding Author*: Associate Professor Jessica Heerde, Principal Research Fellow, Department of Paediatrics, The University of Melbourne, Royal Children’s Hospital Campus, 50 Flemington Road, Parkville Victoria 3052. Phone: +61 407 339 319. Email: [jessica.heerde@unimelb.edu.au](mailto:jessica.heerde@unimelb.edu.au)

**Statements and Declarations**

*Availability of data and materials*: The datasets analysed during the current study are not publicly available but may be available from the corresponding author on reasonable request via the Melbourne Children’s LifeCourse Initiative (https://lifecourse.melbournechildrens.com/).

*Competing interests*: The authors declare that the research was conducted in the absence of any commercial or financial relationships that could be construed as a potential conflict of interest. JWT is a director of the not-for-profit company Communities That Care Ltd that distributes the Communities That Care youth survey in Australia. All other authors declare no conflicts of interest/competing interest.

*Funding*: The author(s) disclose receipt of the following financial support for the research, authorship, and/or publication of this article. A/Prof Heerde receives salary and research support from a National Health and Medical Research Council (NHMRC) Investigator Grant (GNT2007722). She holds a Dame Kate Campbell Fellowship awarded by the Faculty of Medicine, Dentistry and Health Sciences (MDHS) at The University of Melbourne. Her early work on this study was supported by a Momentum Fellowship from the MDHS at the University of Melbourne, the Department of Paediatrics at the University of Melbourne and the Centre for Adolescent Health, Royal Children’s Hospital. Dr Monika Raniti is supported by the NHMRC Centre of Research Excellence in Driving Global Investment in Adolescent Health (GNT1171981) and the ALIVE National Centre for Mental Health Research Translation (NHMRC GNT2002047). Professor Patton was supported by a NHMRC Investigator Grant (GNT1196999). Professor Susan Sawyer is supported by the Geoff and Helen Handbury Chair of Adolescent Health, Department of Paediatrics, The University of Melbourne and an NHMRC Investigator Grant (GNT1196999). The authors are grateful for the financial support of the National Institute on Drug Abuse (R01DA012140), National Institute on Alcoholism and Alcohol Abuse (R01AA025029), NHMRC (GNT491241, GNT594793, GNT1047902) and Australian Research Council (DP109574, DP0663371, DP0877359). The content is solely the responsibility of the authors and does not necessarily represent the official views of the funders. For the purposes of open access, the author has applied a CC BY public copyright licence to any Author Accepted Manuscript version arising from this submission.

*Authors’ contributions*: JAH contributed to project conception, design, analysis, interpretation of results, and drafting and editing the manuscript; JAB contributed to project conception, design, analysis, interpretation of results, and editing the manuscript; GJM contributed to data analysis, interpretation of results, and editing the manuscript. MR contributed to editing the manuscript. GCP contributed to project conception. JWT contributed to interpretation of results and editing the manuscript. SMS contributed to interpretation of results and editing the manuscript. All authors have read and approved the manuscript.

*Acknowledgements*: We acknowledge and pay respect to the passing of our cherished colleague, mentor, and friend Professor George C Patton. The authors wish to express their appreciation and thanks to project staff and participants for their valuable contributions to the project.
